# Protonophore activity of short‐chain fatty acids induces their intracellular accumulation and acidification

**DOI:** 10.1002/1873-3468.70064

**Published:** 2025-05-05

**Authors:** Muwei Jiang, Frans Bianchi, Geert van den Bogaart

**Affiliations:** ^1^ Department of Molecular Immunology Groningen Biomolecular Sciences and Biotechnology Institute, University of Groningen Groningen The Netherlands

**Keywords:** butyrate, cellular pH, histone acetylation, inflammatory cytokines, SCFAs

## Abstract

Short‐chain fatty acids (SCFAs), produced by dietary fiber fermentation in the colon, play essential roles in cellular metabolism, with butyrate notably modulating immune responses and epigenetic regulation. Their production contributes to an acidic colonic environment where protonated SCFAs permeate membranes, leading to intracellular acidification and SCFA accumulation. Using our method to measure intracellular pH, we investigated how extracellular pH influences butyrate‐induced acidification and immunomodulatory effects in human macrophages. Our data show that butyrate accumulates and acidifies cells at acidic extracellular pH due to the permeability of its protonated form. While inflammatory cytokine production was mildly influenced by extracellular pH, butyrate‐induced histone acetylation exhibited a pH dependence, underscoring the importance of considering extracellular pH when assessing the SCFA's functions.

## Graphical abstract

The protonated form of butyrate, as well as other short‐chain fatty acids (SCFAs), is membrane permeable. In acidic extracellular environments, this can lead to intracellular accumulation of SCFAs and cytosolic acidification. This phenomenon will be particularly relevant in acidic environments such as the large intestine or tumor microenvironments.

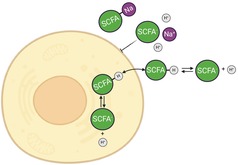

## Abbreviations

**GPCR**, **G protein‐coupled receptor**

**IBD**, inflammatory bowel disease

**IL**, interleukin

**LPS**, lipopolysaccharide

**MCT**, monocarboxylate transporter

**SCFA**, short‐chain fatty acid

**TME**, tumor microenvironment

**TNF‐α**, tumor necrosis factor alpha

Short‐chain fatty acids (SCFAs) are a group of fatty acids consisting of one to six carbon atoms, primarily produced by the fermentation of dietary fibers by gut microbiota in the colon [1]. The most abundant SCFAs are acetate, propionate, and butyrate which are produced in the human proximal colon at 70–140 mm concentration [2]. SCFAs serve not only as a key energy source for colonocytes but also as signaling molecules. Among SCFAs, butyrate has attracted significant attention for its diverse regulatory functions [3]. For example, butyrate promotes intestinal barrier integrity by enhancing tight junction formation and suppressing pro‐inflammatory pathways [4]. Thereby, butyrate shows therapeutic potential in conditions like inflammatory bowel disease (IBD) and colorectal cancer [5]. Currently, butyrate is recognized for exerting its physiological effects through multiple mechanisms, including activation of G protein‐coupled receptor (GPCR) signaling pathways, PPAR‐γ activation, and epigenetic modifications [6, 7, 8]. These processes ultimately lead to changes in gene expression, thereby altering biological functions.

Our previous study demonstrated that butyrate exerts concentration‐dependent effects on human macrophages. At low concentrations, butyrate exhibits anti‐inflammatory properties, suppressing pro‐inflammatory cytokines such as tumor necrosis factor alpha (TNF‐α), while high concentrations of butyrate exhibit pro‐inflammatory effects, promoting the production of TNF‐α and interleukin 1 beta (IL‐1β) and altering macrophage polarization. These findings underscore the complexity of butyrate's role in immune regulation, which may be attributed to differences in intracellular signaling pathways or variations in the external factors, such as modulation by the gut's acidic environment.

The intestinal pH environment is shaped by the dynamic interplay between microbial SCFA metabolism and host physiological processes [9]. As a result, the colon is acidic and the pH ranges from approximately 5.7 in the proximal colon to 6.6 in the distal colon [10]. SCFAs have a pKa below this pH range, meaning that they will mostly be at their nonprotonated form. At a given external pH (pH^E^), the concentration of a species of protonated (i.e., membrane permeable) SCFA [H‐SCFA] is given by:

(1) $$10^{-\mathrm{pKa}}=10^{-{\mathrm{pH}}^{E}}\frac{{\left[\text{SCFA}\right]}^{E}-\left[H-\text{SCFA}\right]}{\left[H-\text{SCFA}\right]}$$

With [SCFA]^E^ the total concentration of extracellular SCFA. For example, the pKa of butyrate is 4.82 [11], and at pH 5.7, only about 11% will be in its protonated form [12]. Small changes in extracellular pH significantly impact the ratio of protonated to unprotonated SCFAs and can be expected to influence their biological activity [13].

**Fig. 1:**
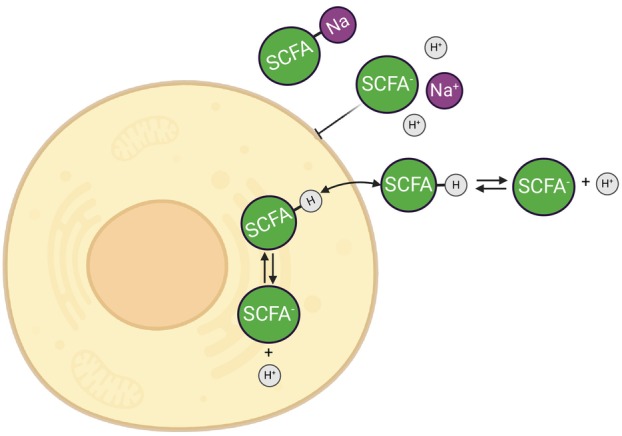
Permeable butyrate [But‐H] induces more protons into the cells, leading to intracellular acidification.

However, the cytosol of cells is neutral, around pH 7.2 [14], and the protonated form of SCFAs is highly membrane permeable [13, 15]. Therefore, the protonated form freely diffuses into the cells. Once inside the more basic cytosol, protonated SCFAs release protons, resulting in SCFA accumulation and intracellular acidification (Fig. 1). Thus, the pH gradient between the extracellular and intracellular environments facilitates SCFA accumulation within cells, contributing to intracellular acidification [16]. Of course, the cells will metabolize and/or esterify the SCFAs and also counter the acidification. However, given that (i) SCFAs are present in 70–140 mm concentration in the colon [2], (ii) protonated SCFAs can readily diffuse over membranes [12, 16], and (iii) the volume of the lumen of the colon is very large compared to the intracellular volume of epithelial cells, it can still be expected to lead to acidification inside the cells, as shown previously for yeast cultured in acidic medium [17, 18, 19].

At equilibrium conditions, where the intracellular and extracellular pH are stable, the SCFA accumulation inside cells can be estimated. From Eq. (1) it follows that:

(2) $$\left[H-\text{SCFA}\right]=\frac{{\left[\text{SCFA}\right]}^{E}}{10^{{\mathrm{pH}}^{E}-\mathrm{pKa}}+1}$$

Since the extracellular compartment is much larger than the intracellular compartment and protonated SCFA is membrane permeable [12, 15], it can be assumed that at equilibrium [H‐SCFA] is equal to the intracellular concentration of protonated SCFA. Therefore, at equilibrium, the total concentration of SCFA within the cytosol of the cells [SCFA]^I^ will be:

(3) $${\left[\text{SCFA}\right]}^{I}=\frac{10^{{\mathrm{pH}}^{I}-\mathrm{pKa}}+1}{10^{{\mathrm{pH}}^{E}-\mathrm{pKa}}+1}{\left[\text{SCFA}\right]}^{E}$$

With pH^I^ the pH in the cytosol. Thus, in case the cytosol is more basic than the extracellular environment (pH^I^ > pH^E^), SCFAs will at equilibrium be present at higher concentrations in the cytosol.

We recently developed a method for the determination of intracellular pH with high accuracy [20]. Therefore, we used this technique to determine the effect of butyrate on the intracellular pH. Given the acidic environment of the gut [10], it is essential to understand how pH modulates butyrate's biological activity. Since we recently found that butyrate exerts profound immunomodulatory properties in cultured human macrophages, we also assessed the effects of butyrate on the production of the inflammatory cytokine TNF‐α and IL‐1β at different extracellular pH. This study builds on our previous findings by exploring the interplay between butyrate, pH, the immunomodulatory function, and histone acetylation in macrophages. By investigating how pH modulates the epigenetic and immunological effects of butyrate, we aim to elucidate the mechanisms underlying its concentration‐dependent functions in health and disease.

## Materials and methods

### Cell lines and cell culture

HeLa cells (RRID: CVCL_0030) were cultured in Dulbecco's modified Eagle's medium (DMEM, Gibco, Thermo Fisher Scientific, Waltham, MA, USA, 2858843) supplemented with 10% fetal bovine serum (FBS, Hyclone, Logan, UT, USA 10309433), 1% antibiotic‐antimitotic solution (AA, Gibco, 15240062), and 1% l‐glutamine (Gibco, 15430614) at 37 °C in a humidified atmosphere containing 5% CO_2_. The cell line was authenticated by ATCC (Manassas, VA, USA) through their human STR profiling cell authentication service. All experiments were performed with mycoplasma‐free cells.

Peripheral blood mononuclear cells (PBMC) were isolated from buffy coats of human blood donors using ficoll density gradient centrifugation. The buffy coats were anonymized and provided by the Dutch blood bank. All blood donors were informed about the research and granted their consent to the blood bank. Ethical approval was not required under the Dutch Medical Research Involving Human Subjects Law, as samples were fully anonymized and donors did not undergo additional procedures. CD14^+^ monocytes were isolated from PBMCs using CD14 MicroBeads (Miltenyi Biotec, Bergisch Gladbach, Germany, 130‐050‐201). Human peripheral blood monocyte‐derived macrophages were differentiated in Ultra‐low adherent 6‐well plates (Corning, Corning, NY, USA, 3471) with 2 mL of RPMI 1640 medium (Gibco, 11875093) supplemented with 10% FBS, 1% AA, 1% l‐glutamine, and M‐CSF (R&D Systems, Minneapolis, MN, USA, 216‐MC, 100 ng·mL^−1^) at 37 °C and 5% CO_2_ for 7 days. On day 4, the macrophages were replenished with an additional 1 mL of medium containing 50 ng·mL^−1^ M‐CSF. The study methodologies conformed to the standards set by the Declaration of Helsinki.

### Reagents

Buffers with defined pH values for generating the calibration curve were prepared as previously described [1]. The calibration buffers consisted of 125 mm KCl, 25 mm NaCl, and either 25 mm N‐[2‐hydroxyethyl]‐piperazine‐N‐[2‐ethanesulfonic acid] (HEPES) for pH 7.0, 7.5, and 8.0, or 25 mm 2‐[N‐morpholino]ethanesulfonic acid (MES) for pH 3.5, 4.77, 5.5, 6.0, and 6.5. Each buffer was adjusted to the desired pH using 1 m NaOH or 1 m HCl at 37 °C. Sodium butyrate (Merck, Darmstadt, Germany, 303 410) was dissolved in the prepared buffers at the defined pH values.

### Transfection

GPI‐RpHLuorin2 (Addgene, Watertown, MA, USA, 1717121, RpHLuorin2 empty vector (Addgene, 180227), and LAMP1‐RpHLuorin2 (Addgene, 171720) were transfected into HeLa cells using the Neon Transfection System (Invitrogen, Waltham, MS, USA, MPK10096). For electroporation, 2 μg of DNA was used per 1 × 10^5^ cells, with two pulses of 35 ms at 1000 V. Following transfection, the cells were seeded into a 4‐compartment CELLview Cell Culture Dish (Greiner Bio‐One, Kremsmuenster, Austria) at a density of 2.5 × 10^4^ cells per well and incubated at 37 °C with 5% CO₂ for 48 h.

### Confocal microscopy

After 48 h of incubation, the medium was removed and replaced with defined pH buffers with or without butyrate. Imaging was performed using a Zeiss LSM800 microscope with a 40× objective lens. The acquired images were analyzed with FIJI ImageJ, and the 405/488 nm intensity ratios were calculated automatically using a custom macro provided in the Supplementary Information.

### Enzyme‐linked immunosorbent assay (ELISA)

PBMC‐differentiated macrophages were seeded into the flat‐bottom 96‐well plate (Corning, 3596) at a density of 100 000 cells per well. The cells were treated for 24 h with or without 1 μg·mL^−1^ LPS, 20 ng·mL^−1^ IFN‐γ, and sodium butyrate in defined pH buffers. TNF‐α and IL‐1β levels were measured using ELISA kits (Invitrogen, 88‐7346, 88‐7261) following the manufacturer's protocols.

### Western blotting

PBMC‐differentiated macrophages (1 × 10^6^ cells per condition) were treated with 1 μg·mL^−1^ LPS, 20 ng·mL^−1^ IFN‐γ, and either 1 mm or 10 mm sodium butyrate in pH 6.5, 7.0, or 8.0 buffers for 24 h. After treatment, the cells were scraped and lysed using RIPA Lysis and Extraction Buffer (Thermo Fisher Scientific, Waltham, MA, USA, 89 900) supplemented with a protease inhibitor (Roche, Basel, Switzerland, 04693159001). Protein concentrations were quantified using the Micro BCA Protein Assay Kit (Thermo Fisher Scientific). Equal amounts of protein lysate from each condition were loaded onto 4–20% Mini‐Protein TGX precast gels (Bio‐Rad, Hercules, CA, USA) and electrophoresed at 100 V, followed by transfer onto PVDF membranes at 90 V for 60 min. The membranes were blocked in 5% BSA (Thermo Fisher Scientific, BP9702) at room temperature for 1 h. Following blocking, the membranes were washed three times with 0.01% TBST and incubated overnight at 4 °C with the following primary antibodies: polyclonal rabbit H3AcK4 + 9 + 14 + 18 + 23 + 27 (Ac‐H3Kn) antibody (1:500, Abcam, Cambridge, UK, ab300641), monoclonal mouse Histone H3 antibody (1:100, Santa Cruz Biotechnology, Dallas, TX, USA, sc‐517 576), and monoclonal mouse GAPDH antibody (1:1000, Santa Cruz Biotechnology, sc‐365 062). The next day, the membranes were washed three times with 0.01% TBST and incubated with secondary antibodies, Donkey anti‐Rabbit IgG IRDye680 (1:5000, LI‐COR, Lincoln, NE, USA, 926‐32 223) or Donkey anti‐Mouse IgG IRDye680 (1:5000, LI‐COR, 926‐32 222), for 1 h at room temperature. After three additional washes with TBST, the membranes were scanned using the Odyssey CLx Infrared Imaging System and analyzed with Image Studio software (LI‐COR).

### Statistical analysis

The pH values were determined using the equation derived from the calibration curve as described previously [21, 22]. Statistical analysis was performed on GraphPad software. Statistical tests and significance levels (*P*‐values) are stated in the figure legends.

## Results

### Butyrate accumulates in the cytosol in an acidic microenvironment

**Fig. 2:**
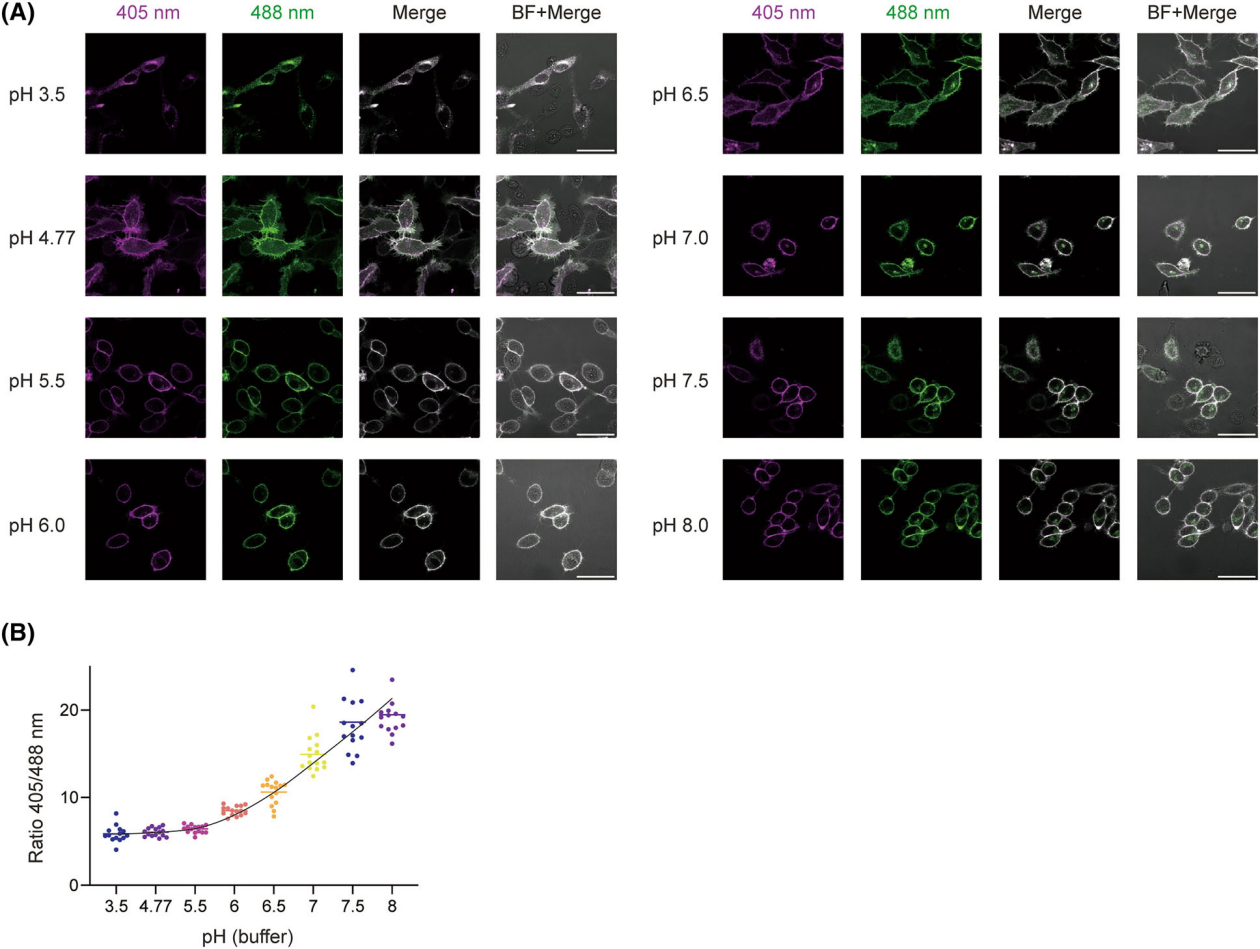
Calibration of RpHLuorin2 in HeLa cells expressing GPI‐RpHLuorin2. Hela cells were transfected with GPI‐anchored RpHLuorin2. (A) Representative confocal laser scanning microscopy images of HeLa cells expressing GPI‐RpHLuorin2 in defined calibration buffers. Images show 405 nm excitation (magenta) and 488 nm excitation (green). Scale bars: 50 μm. (B) Calibration curve for the ratio 405/488 nm based on panel A (*n* = 15 cells; error bar represent mean). All calibration curves for the other independent experiments are shown in Fig. S1.

We determined how butyrate affects the cytosolic pH by measuring the intracellular pH in the presence and absence of butyrate. We first calibrated the GPI‐anchored RpHLuorin2 probe in HeLa cells, as we described previously [20]. GPI‐anchored proteins predominantly localize at the plasma membrane; hence, the pH that the RpHLuorin2 probe is exposed to can be simply altered by changing the extracellular pH. Following transfection, cells were imaged using confocal microscopy with alternating excitation at 405 nm and 488 nm in defined pH buffers ranging from pH 3.5 to 8.0 (Fig. 2A). We selected this broad pH range because it corresponds to the pH range that cells are exposed to in the intestine and hypoxic tumors [10, 23]. The fluorescence intensity ratios (405/488 nm) were calculated for each pH condition, and calibration curves were generated for each experiment (Fig. 2B; Fig. S1A,B). We then used these calibration curves for quantifying intracellular pH changes in subsequent experiments.

For selecting cells with similar expression levels, we kept the microscope settings the same for all experiments and selected cells with similar fluorescence intensities based on the microscopy images. Expression levels do not significantly influence our results, as we previously showed using the same RpHLuorin2 constructs that the fluorescence intensities do not correlate significantly with the fluorescence lifetimes [20], arguing against notable contributions of background fluorescence or self‐quenching effects at the measured expression levels. This was further confirmed by performing experiments in technical triplicates (i.e., with three batches of transfected cells) to exclude batch‐to‐batch variation of transfection efficiencies.

**Fig. 3:**
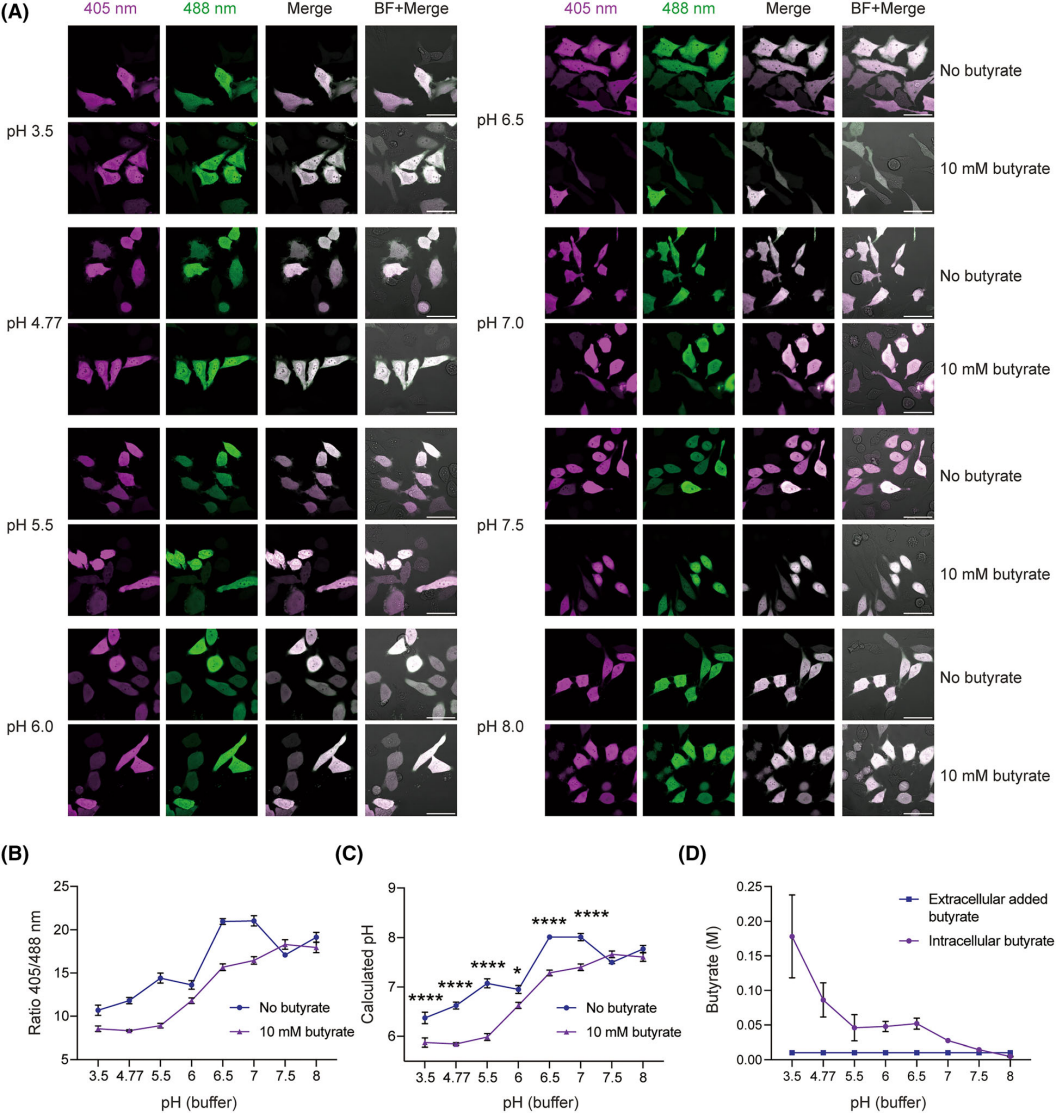
Butyrate causes intracellular acidification. Hela cells were transfected with the RpHLuorin2 empty vector. (A) Representative confocal laser scanning microscopy images of HeLa cells expressing RpHLuorin2 in defined pH buffers with and without 10 mm butyrate. Images show 405 nm excitation (magenta) and 488 nm excitation (green). Scale bars: 50 μm. (B) Determination of the ratio 405/488 nm based on panel A (*n* = 15 cells; error bar represents means ± SEM; other independent experiments are shown in Fig. S2A,B). (C) Calculation of pH values based on panel B and the calibration curve from panel B (*n* = 15 cells; two‐way ANOVA with a Bonferroni's multiple comparisons test, *P < 0.05; ****P < 0.0001; error bar represents means ± SEM; other independent experiments are shown in Fig. S2C,D). (D) Calculation of intracellular butyrate equilibrium concentration based on panel C and the pH value from panel C; *n* = 3 independent experiments.

To investigate the effects of butyrate on intracellular pH, we next transfected HeLa cells with a plasmid coding for nonconjugated RpHLuorin2. In this case, RpHLuorin2 locates in the cytosol and nucleus of the cells, hence allowing for the determination of the pH in these compartments. These cells were imaged under the same conditions as we used for the GPI‐RpHLuorin2 calibration, in defined pH buffers with or without 10 mm butyrate (Fig. 3A). This butyrate concentration was selected as it is present at this high concentration in the colon of humans [10], so enterocytes and intestinal cancer cells will be exposed to this concentration. Moreover, many *in vitro* studies use butyrate in the mm concentration range [24, 25, 26, 27].

Fluorescence intensity ratios (405/488 nm excitation) were calculated (Fig. 3B; Fig. S2A,B), and intracellular pH values were determined using the calibration curves (Fig. 3C; Fig. S2C,D). As expected, butyrate induces significant intracellular acidification within the pH range of 3.5 to 7.0. This finding shows butyrate's potential to lower the intracellular pH when the extracellular pH is acidic. In addition, based on Eq. (3), at acidic concentrations, butyrate (and other SCFA species) can be expected to accumulate within the cells (Fig. 3D).

Given the essential role of lysosomes in cellular metabolism and signaling [28], we also examined whether butyrate affects lysosomal pH. Lysosomes are very acidic organelles, with a pH ranging between 4.5 and 5.5 [29], and SCFAs are thus not expected to accumulate in these compartments. We transfected HeLa cells with LAMP1‐RpHLuorin, a lysosome‐targeted version of the pH‐sensitive probe. Cells were imaged using the same excitation channels (405 nm and 488 nm) and in the same pH buffers as before, with or without 10 mm butyrate (Fig. S3A). Fluorescence ratios were calculated (Fig. S3B,C), and lysosomal pH was determined using the calibration curve (Fig. S3D,E). As expected, and unlike its effects on cytosolic pH, butyrate did not significantly alter lysosomal pH under any of the tested conditions, which indicates that lysosomal acidification remains stable in the presence of butyrate.

### pH affects slightly influences butyrate's effects on TNF‐α production by macrophages

Based on our previous observations of butyrate's concentration‐dependent effects on human macrophages, we explored whether extracellular pH influences TNF‐α and IL‐β production. We treated human macrophages derived from peripheral blood monocytes with different concentrations of butyrate in media adjusted to physiological pH values of 6.5, 7.0, 7.5, and 8.0. We did not include more acidic pH values, as they negatively affect cell viability. The cells were stimulated with the pathogenic stimulus lipopolysaccharide (LPS) and the inflammatory cytokine interferon gamma (IFN‐γ) for induction of cytokine production.

**Fig. 4:**
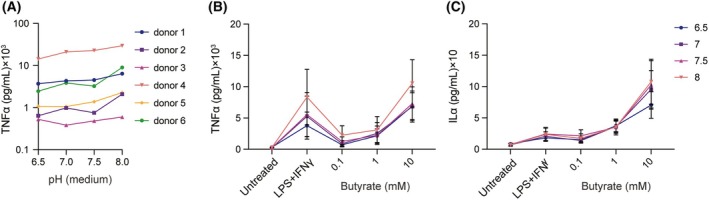
pH affects inflammatory cytokine production of human monocyte‐derived macrophages. (A) Human peripheral blood monocyte‐derived macrophages were stimulated for 24 h with LPS and IFN‐γ in defined pH medium; TNF‐α production was determined by ELISA (*n* = 6 donors). (B, C) Human peripheral blood monocyte‐derived macrophages were stimulated for 24 h with LPS, IFN‐γ, and the indicated concentrations of Na‐butyrate in defined pH medium. TNF‐α (B) and IL‐1β (C) production were determined by ELISA (*n* = 6 donors; the error bars represents means ± SEM).

We first determined how pH affected cytokine production in the absence of butyrate using ELISA for TNF‐α. For 6 donors, a more basic pH consistently resulted in an increased production of TNF‐α, whereas production was reduced by acidic pH (Fig. 4A). Among the donors, the variation in cytokine production various more than 100‐fold, as is well known for cytokine production [30]. This result aligns with previous findings indicating that pro‐inflammatory markers in macrophages are suppressed in the acidic tumor microenvironment [23, 31].

Next, we determined the effects of butyrate at different extracellular pH. As we reported previously, our results demonstrated that 0.1 and 1 mm butyrate inhibited TNF‐α production at all tested pH conditions, whereas 10 mm butyrate increased production of TNF‐α and IL‐1β (Fig. 4B,C). These findings indicate that although pH affects the production of inflammatory cytokines by macrophages, the immunomodulatory effects of butyrate are not pH dependent. At a broad physiological pH range, low concentrations of butyrate still retain their anti‐inflammatory properties, while high concentrations of butyrate remain pro‐inflammatory.

**Fig. 5:**
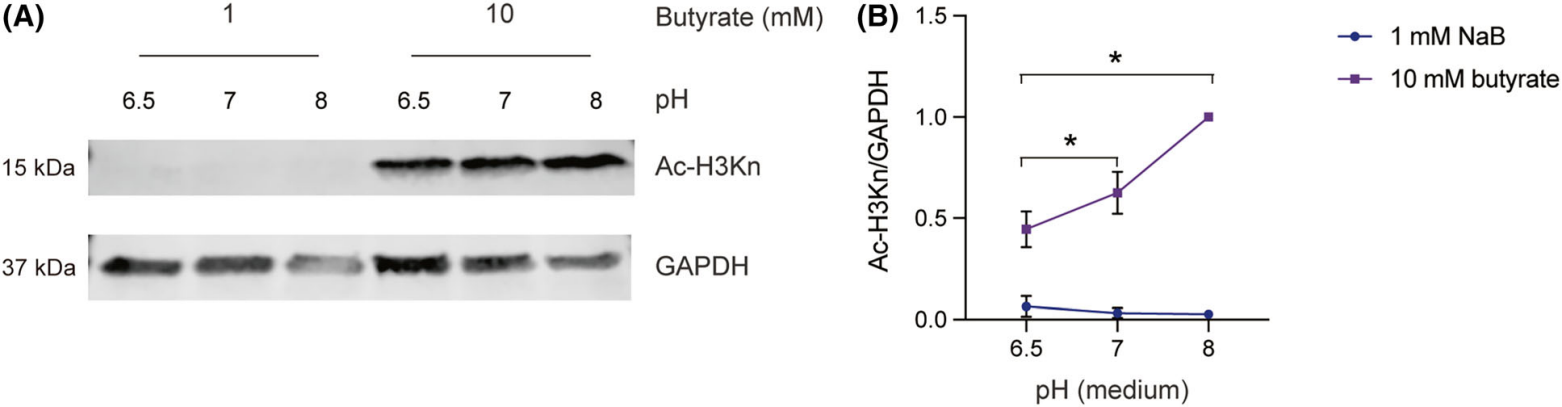
Butyrate increases histone acetylation at alkaline pH condition. Human peripheral blood monocyte‐derived macrophages were stimulated for 24 h with LPS, IFN‐γ, and the indicated concentrations of Na‐butyrate in defined pH medium. (A) Western blots showing histone acetylation detected with antibodies recognizing (Ac‐)H3K4 + 9 + 18 + 23 + 27 (Ac‐H3Kn) acetylation. (B) Quantification of Ac‐H3Kn levels normalized to GAPDH (*n* = 3 donors; one‐way ANOVA with a Dunnett's multiple comparisons test, *P < 0.05; the error bars represent means ± SEM; the complete blots for all donors are shown in Fig. S4).

From previous research by us and other laboratories [32], it is known that butyrate increases histone acetylation in human macrophages in a concentration‐dependent manner. Since we found that an acidic extracellular pH results in the accumulation of butyrate, we also determined whether this would affect histone acetylation. We treated macrophages with 1 mm and 10 mm butyrate in buffers adjusted to pH 6.5, 7.0, and 8.0. Then, we performed western blot analysis to detect the histone acetylation. Our results showed that 1 mm butyrate did not induce detectable histone acetylation under any pH condition. However, treatment with 10 mm butyrate significantly increased histone acetylation at all extracellular pH conditions (Fig. 5A; Fig. S4), consistent with our previous findings. Notably, histone acetylation levels were higher at pH 8.0 compared to pH 6.5 and 7.0 (Fig. 5B), indicating a pH‐dependent iof butyrate's effects on histone acetylation.

## Discussion

The pH of the intracellular environment plays a critical role in maintaining cellular homeostasis, potentially impacting processes such as immune responses and gene expression [33]. In the human body, the extracellular pH varies across different regions and is especially acidic in the gut due to the anaerobic fermentation of dietary fibers [10]. Not only does this result in acidification, but the SCFAs that are produced from the fibers are widely recognized for their immunomodulatory properties [2]. However, SCFAs are membrane permeable in their protonated form [15] and if the extracellular pH is lower than the intracellular pH, this can lead to SCFA accumulation and acidification of cells. In this study, we investigated the changes in intracellular pH in the presence of butyrate under varying external pH conditions. Additionally, we examined how the production of TNF‐α and IL‐1β is influenced by butyrate and pH.

Our data suggest that butyrate induces intracellular acidification when the extracellular pH is lower than that of the cytosol, at a pH range of 3.5 to 7.0. Thus, at acidic extracellular pH values, the protonated form of butyrate freely diffuses into cells, leading to an accumulation of butyrate and the release of protons in the neutral cytosol, thereby contributing to acidification. Our previous studies showed that low concentrations of butyrate inhibit inflammatory cytokine production by cultured macrophages, while high concentrations promote these, and that butyrate increases histone acetylation in a concentration‐dependent manner. However, our data show that at least between pH 6.5 and 8, the extracellular pH does not affect butyrate's effects on cytokine production. In contrast, the histone acetylation induced by butyrate is influenced by external pH conditions, as we found increased levels of histone acetylation at basic pH.

In our study, we focused on butyrate given its strong immunomodulatory effects, but other SCFAs can also be expected to accumulate in the cells and acidify the cytosol in acidic extracellular environments. Studies using liposomes made of lipid extracts from mammalian cells and synthetic lipids have shown that the protonated forms of formate, acetate, propionate, and lactate are membrane permeable [12, 15, 16, 17].

In the human intestine, the pH starts at approximately 5.7 in the proximal colon and gradually increases to around 6.6 in the distal colon [10]. Similarly, the SCFA concentration is highest in the proximal colon, reaching up to 140 mm and gradually decreases to around 90 mm in the distal colon [10]. Under such conditions, our findings suggest that the accumulation of different species of SCFAs within enterocytes could lead to intracellular concentrations in the molar level, potentially causing severe acidification of the cytosol and osmotic stress, which would result in disruption of physiological processes and likely cause cytotoxicity [33]. Therefore, enterocytes must have evolved mechanisms to prevent such outcomes. These likely include rapid metabolism of SCFAs into acetyl‐CoA for energy production and the use of monocarboxylate transporters (MCTs) to efficiently expel excess SCFAs from the cytosol [34, 35]. These processes are not only critical for maintaining cellular homeostasis but also for supporting the high energy demands of enterocytes, which rely on SCFA metabolism.

The ability of enterocytes to balance SCFA uptake and removal underscores the importance of tight regulation in the gut environment. Disruptions to this balance, such as those caused by altered microbiota composition or gut inflammation, could exacerbate SCFA accumulation and acidification, leading to impaired barrier function and inflammation [36]. We therefore hypothesized that a lower extracellular pH would cause the accumulation of SCFAs and enhance the pro‐inflammatory response in macrophages. However, our findings indicated otherwise. Consistent with our previous report, we observed that low concentrations of butyrate inhibit inflammatory cytokine production, while high concentrations of butyrate promote this, even at low extracellular pH. Furthermore, our results showed that following inflammatory stimulation, a basic pH increased the production of TNF‐α, whereas a slightly acidic pH led to a reduction in TNF‐α levels. This observation aligns with findings that macrophages in the tumor microenvironment (TME) exhibit reduced production of pro‐inflammatory cytokines [23].

We observed that butyrate promotes histone acetylation under basic pH conditions, whereas histone acetylation was lower at acidic conditions. As intracellular pH (pH^i^) decreases, histone deacetylases (HDACs) deacetylate histones to release acetate anions, which are exported along with protons via monocarboxylate transporters (MCTs) to prevent further pH^i^ reduction [37]. Based on our results, while we were unable to detect histone acetylation levels in the absence of butyrate due to the low sensitivity of the antibody, the reduced histone acetylation levels observed under acidic conditions in cells exposed to butyrate, compared to those in an alkaline extracellular pH environment, may be explained by this mechanism. As butyrate accumulates intracellularly at acidic conditions, this finding shows that it is unlikely that the intracellular butyrate concentration is limiting for histone acetylation. Instead, it might be that the signaling of butyrate is pH sensitive, for example, pH‐dependent GPCR binding on the cell surface [13], and that this subsequently affects histone acetylation within the cell. Similarly, pH might affect LPS and/or IFN‐γ signaling, and thereby affect TNF‐α production.

Our findings are likely not only relevant to SCFA production in the colon, but also for cancer. In the tumor microenvironment, the Warburg effect leads to an accumulation of lactic acid, a metabolic by‐product of glycolysis [23]. Similar to butyrate, lactic acid is a SCFA that can reach high concentrations (up to 30 mm) in the TME [23]. Moreover, due to lactate production, the extracellular pH of the TME can reach values as low as 6.0 [23]. This creates an acidic environment that promotes the protonation of lactic acid (pKa = 3.86). Since protonated lactate is also membrane permeable [17], it can be expected to diffuse into the tumor cells and contribute to intracellular acidification, similar to our findings with butyrate. To counteract this, tumor cells employ adaptive mechanisms to regulate intracellular pH [38].

In conclusion, at physiological acidic extracellular pH levels, butyrate and likely other SCFAs can accumulate within cells and induce intracellular acidification. In human macrophages, cytokine production was only mildly influenced by external pH. However, butyrate‐induced histone acetylation is promoted at basic pH. Given the variations in pH and SCFA concentrations across different regions of the human body, studying the interplay between extracellular pH, SCFA concentrations, and intracellular acidification, and linking these findings to the immunological and epigenetic regulatory functions of SCFAs has significant implications for understanding the biological mechanisms of SCFAs in health and disease.

## Author contributions

MJ conducted all experiments and wrote the original draft. GvdB and FB conceptualized the project and edited the draft.

## Peer review

The peer review history for this article is available at https://www.webofscience.com/api/gateway/wos/peer‐review/10.1002/1873‐3468.70064.

## Supporting information


**Data S1.** FIJI macro for automated quantification of the 405/488 nm intensity ratio.
**Fig. S1.** Calibration of RpHLuorin2 by CLSM in HeLa cells expressing GPI‐RpHLuorin2.
**Fig. S2.** Butyrate causes intracellular acidification.
**Fig. S3.** Butyrate does not cause lysosomal acidification.
**Fig. S4.** Butyrate enhances histone acetylation at alkaline pH condition.

## Data Availability

All data are available at the repository Zenodo (DOI: 10.5281/zenodo.14809723).
