# Supplementary material for: Protonophore activity of short‐chain fatty acids induces their intracellular accumulation and acidification
Source: FEBS Lett. 2025 May 5;599(15):2157–66. doi: 10.1002/1873-3468.70064 (PMC12338859; doi:10.1002/1873-3468.70064)
Supplement: Supplementary file 1 — Data S1. FIJI macro for automated quantification of the 405/488 nm intensity ratio. Fig. S1. Calibration of RpHLuorin2 by CLSM in HeLa cells expressing GPI‐RpHLuorin2. Fig. S2. Butyrate causes intracellular acidification. Fig. S3. Butyrate does not cause lysosomal acidification. Fig. S4. Butyrate enhances histone acetylation at alkaline pH condition. [file FEB2-599-2157-s001.docx]

**SUPPLEMENTARY MATERIAL TO:**

**Protonophore activity of short-chain fatty acids induces their intracellular accumulation and acidification**

Muwei Jiang, Frans Bianchi, Geert van den Bogaart^*^

Departments of Molecular Immunology, Groningen Biomolecular Sciences and Biotechnology Institute, University of Groningen, 9747AG, Nijenborgh 7, Groningen, the Netherlands.

*Correspondence to: +31 50 363 5230

g.van.den.bogaart@rug.nl

Contents:

- 4 supplementary figures
- FIJI macro for automated quantification of the 405/488 nm intensity ratio


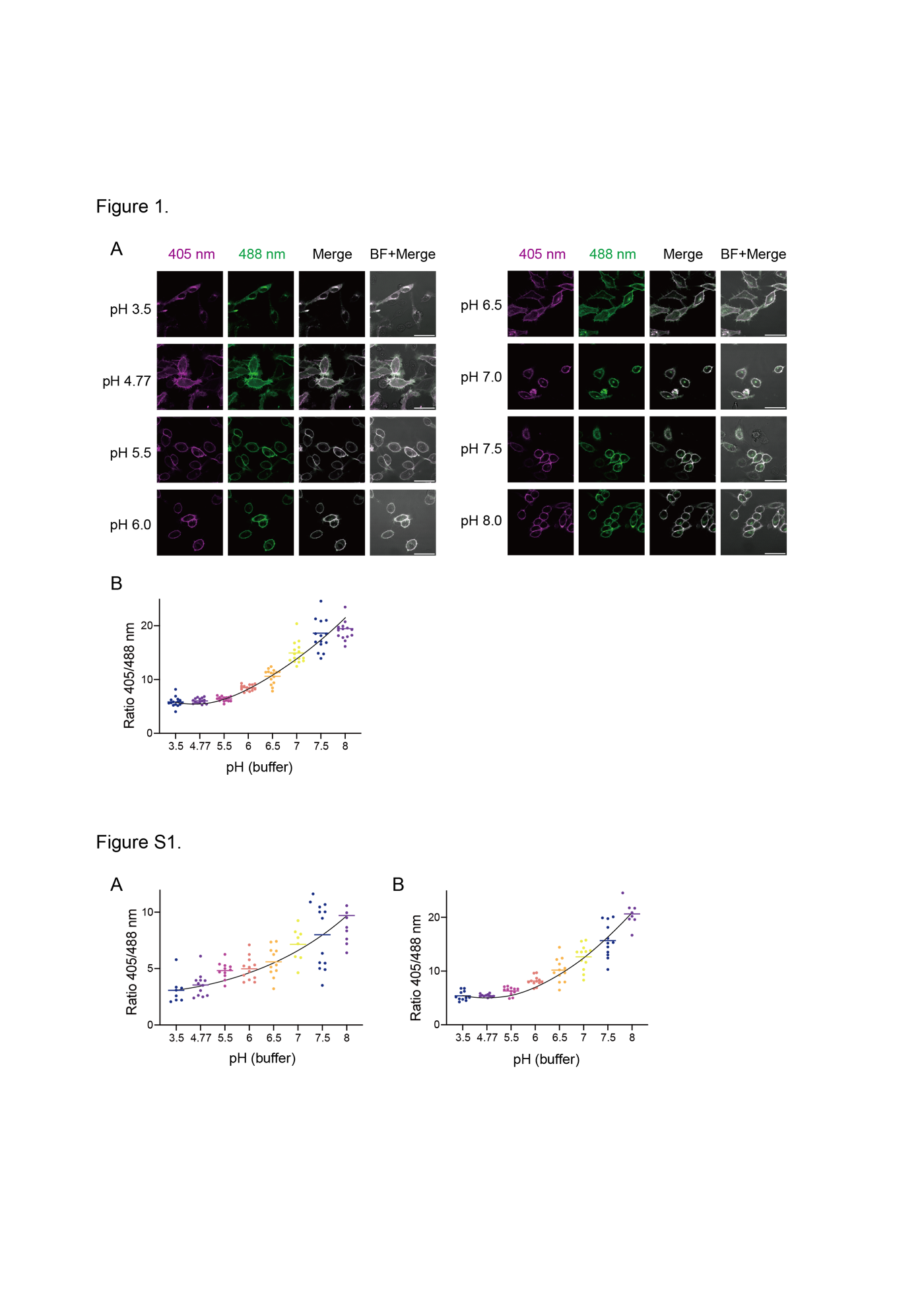


**Fig. S1. Calibration of RpHLuorin2 by CLSM in HeLa cells expressing GPI-RpHLuorin2.**

Hela cells were transfected with the GPI-anchored RpHLuorin2. Confocal laser scanning microscopy was performed of HeLa cells expressing GPI-RpHLuorin2 in defined calibration buffers. The intensity of 405 nm excitation and 488 nm excitation was determined. Calibration curves for the ratios 405/488 nm were calculated based on these confocal images. (A) n = 8 (pH 3.5, 7.0), 12 (pH 4.77, 6.0), 11 (pH 5.5, 6.5, 8.0), 13 (pH 7.5) cells. (B) n = 11 (pH 3.5, 4.77), 12 (pH 5.5, 6.0, 6.5, 7.0, 7.5), 8 (pH 8.0) cells; error bar represent mean.


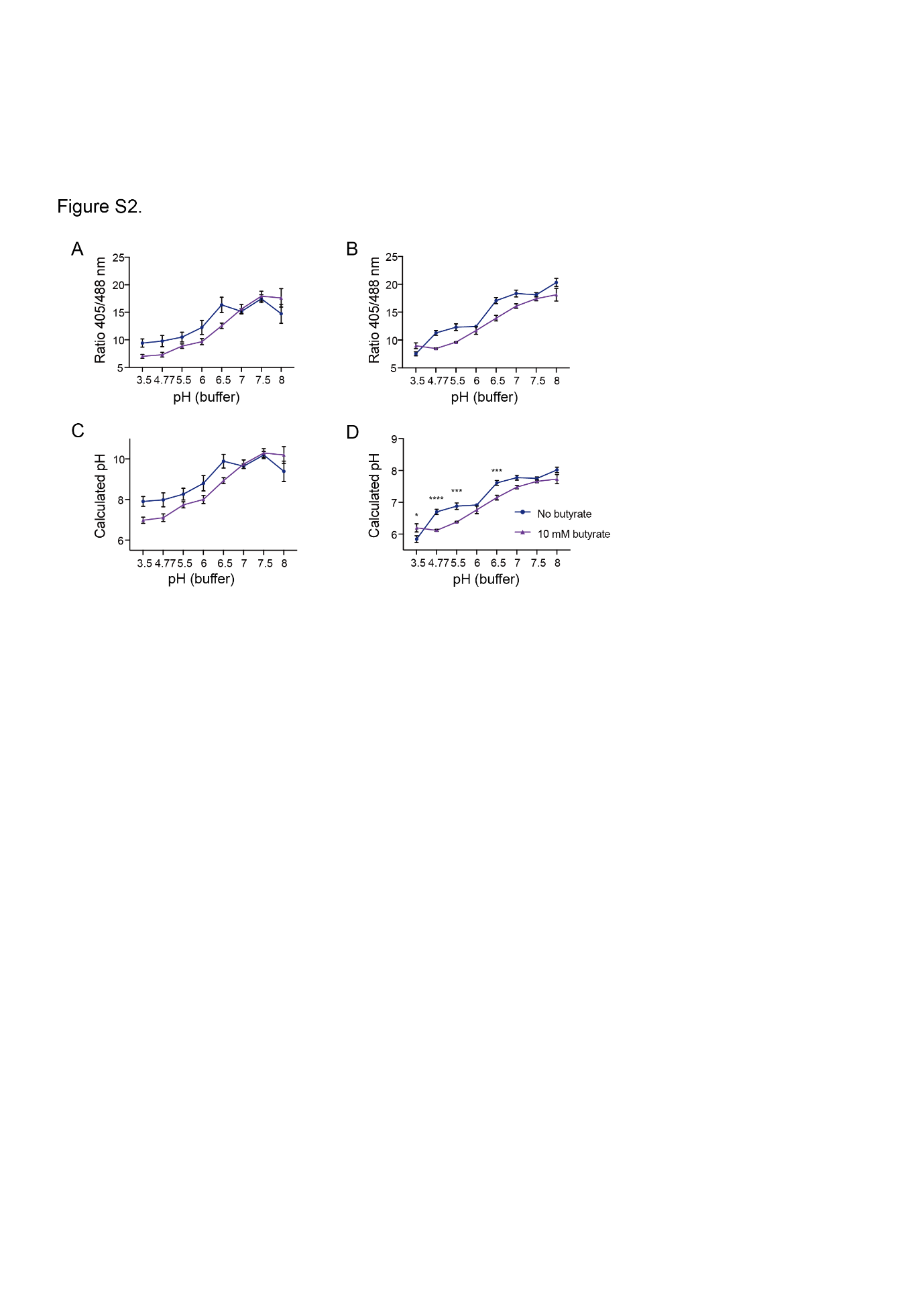


**Fig. S2. Butyrate causes intracellular acidification.** Hela cells were transfected with the RpHLuorin2 empty vector, and stimulated with 10 mM butyrate. Confocal laser scanning microscopy of HeLa cells expressing GPI-RpHLuorin2 was performed in defined pH buffers. (A-B) The ratio of 405 nm excitation and 488 nm excitation intensity was determined on these confocal images. (n > 10 cells; error bar represent means ± SEM). (C-D) Calculation of pH values for the experiment of panel A-B. (n > 10 cells; error bar represent means ± SEM; Two-way ANOVA with a Bonferroni’s multiple comparisons test, *: P < 0.05; ***: P < 0.001; ****: P < 0.0001). (E-F) Calculation of intracellular butyrate equilibrium concentration based on panel C-D and the pH value from panel C-D.


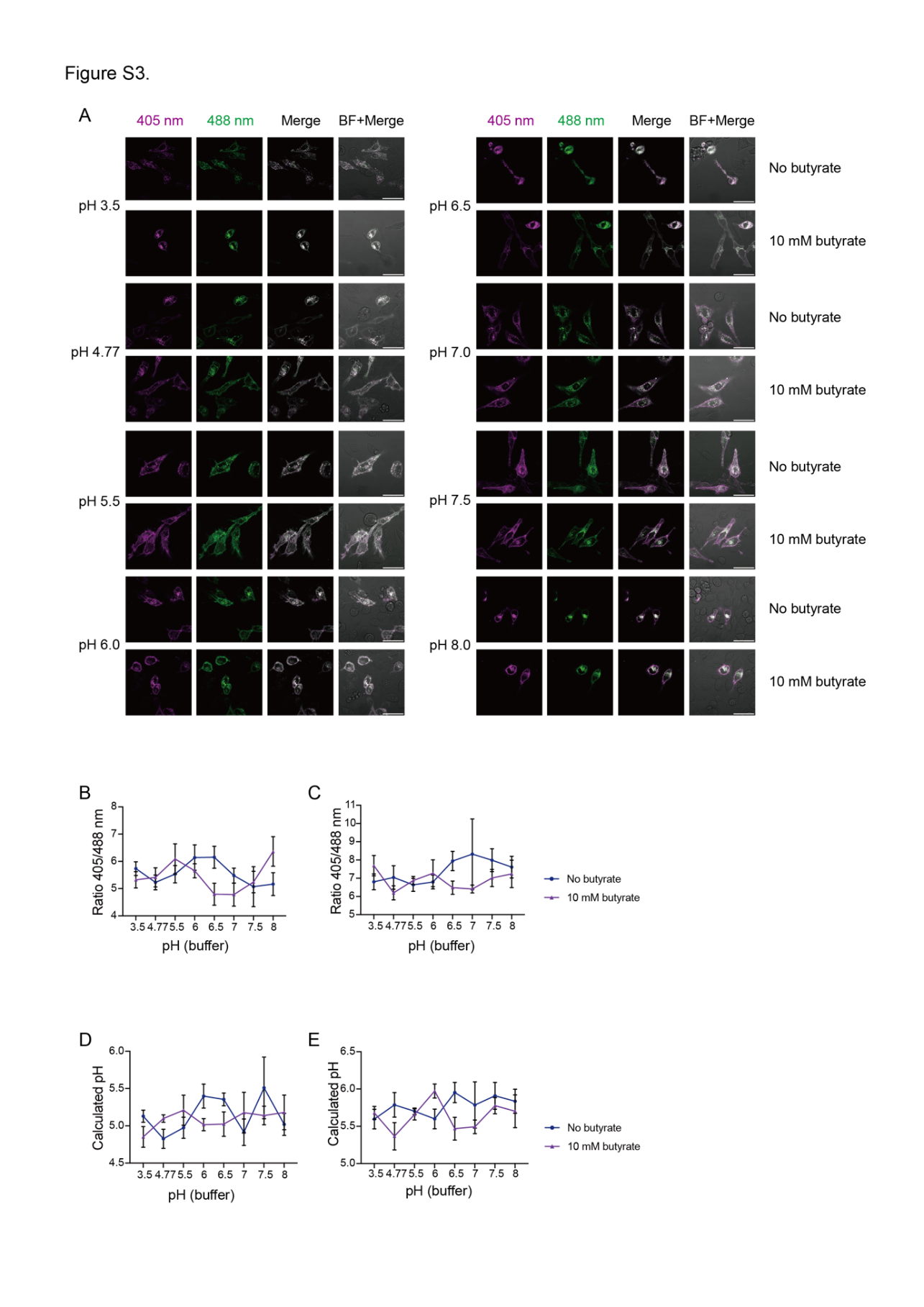


**Fig. S3. Butyrate does not cause lysosomal acidification.** Hela cells were transfected with recombinant LAMP-RpHLuorin2. (A) Representative confocal images of HeLa cells expressing LAMP-RpHLuorin2 in defined pH buffers with 10 mM butyrate. Images showing 405 nm excitation (magenta) and 488 nm excitation (green). Scale bars: 50 μm. (B-C) Determination of ratio 405/488 nm based on panel A. (n >10 cells; error bar represent means ± SEM). (D-E) Calculation of pH values based on panel B-C. (n>10 cells; error bar represent means ± SEM).


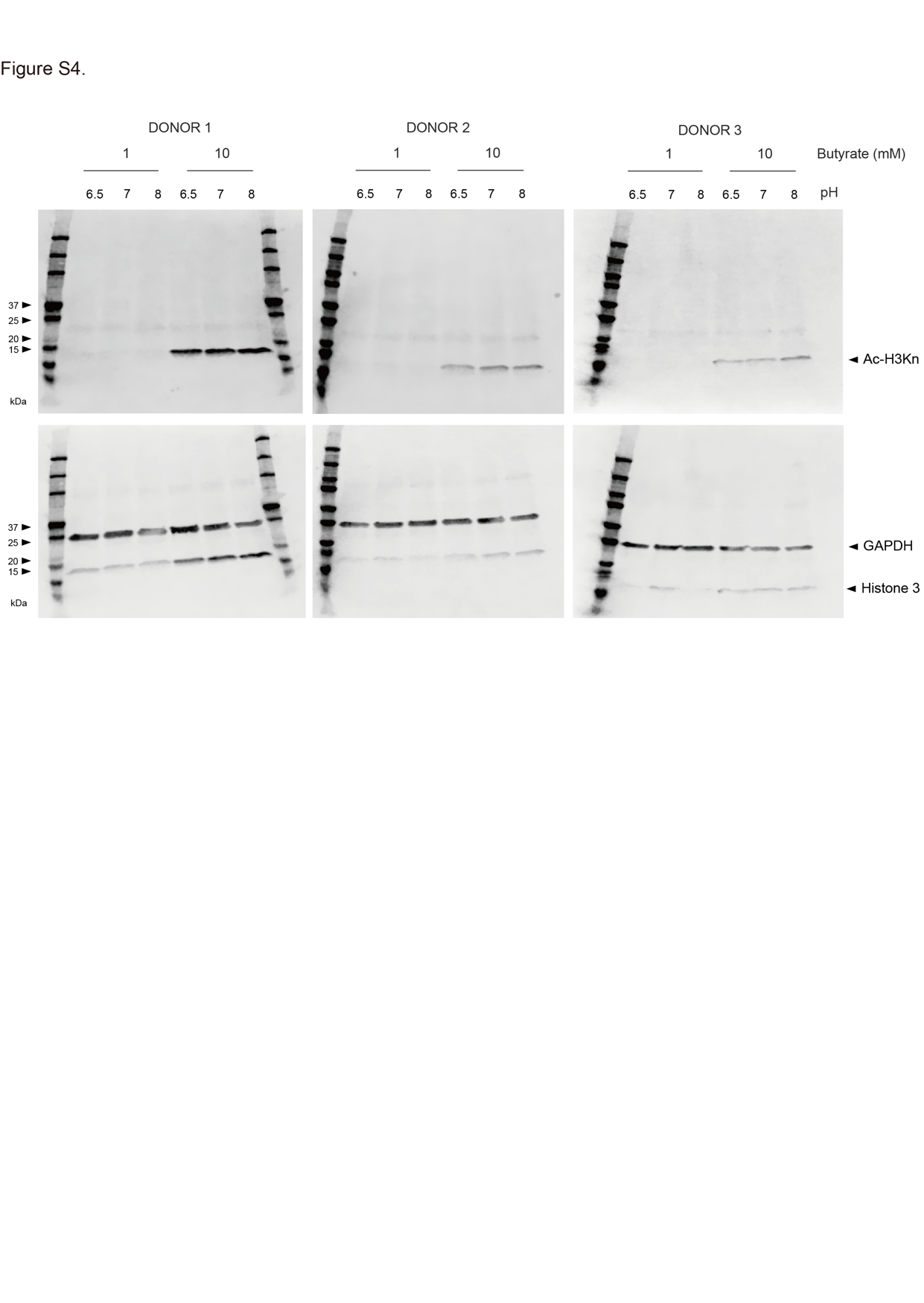


**Fig. S4. Butyrate enhances histone acetylation at alkaline pH condition.**

Human peripheral blood monocyte-derived macrophages were stimulated for 24 hr with LPS, IFN-γ, and the indicated concentrations of Na-butyrate in defined pH media. Complete western blots for 3 donors showing histone acetylation detected with antibodies recognizing (Ac-)H3K4+9+18+23+27 (Ac-H3Kn) acetylation. Blots were stripped and reprobed for GAPDH and total H3.

**Data. S1. FIJI macro for automated quantification of the 405/488 nm intensity ratio**

var OriginalImage = getImageID();

run("Duplicate...", "duplicate channels=1");

run("32-bit");

setAutoThreshold("Default dark");

//run("Threshold...");

run("NaN Background");

var ID488 = getImageID();

var Name488 = getTitle();

var Int488 = getValue("Mean");

close();

selectImage(OriginalImage);

run("Duplicate...", "duplicate channels=1");

run("32-bit");

setAutoThreshold("Default");

//run("Threshold...");

run("NaN Background");

var IDB488 = getImageID();

var NameB488 = getTitle();

var IntB488 = getValue("Mean");

close();

selectImage(OriginalImage);

run("Duplicate...", "duplicate channels=2");

run("32-bit");

setAutoThreshold("Default dark");

//run("Threshold...");

run("NaN Background");

var ID405 = getImageID();

var Name405 = getTitle();

var Int405 = getValue("Mean");

close();

selectImage(OriginalImage);

run("Duplicate...", "duplicate channels=2");

run("32-bit");

setAutoThreshold("Default");

//run("Threshold...");

run("NaN Background");

var IDB405 = getImageID();

var NameB405 = getTitle();

var IntB405 = getValue("Mean");

close();

print(Int405/Int488);

print((Int405-IntB405)/(Int488-IntB488));

selectImage(OriginalImage);

//close();
